# Image-Based Peridynamic Modeling-Based Micro-CT for Failure Simulation of Composites

**DOI:** 10.3390/ma17204987

**Published:** 2024-10-12

**Authors:** Zhuo Wang, Ling Zhang, Jiandong Zhong, Yichao Peng, Yi Ma, Fei Han

**Affiliations:** 1 State Key Laboratory of Structural Analysis, Optimization and CAE Software for Industrial Equipment, Department of Engineering Mechanics, Dalian University of Technology, Dalian 116023, China; 2 School of Engineering, Hangzhou Normal University, Hangzhou 311121, China

**Keywords:** peridynamics, deep-learning, computer tomography, composite material, failure simulation

## Abstract

By utilizing computed tomography (CT) technology, we can gain a comprehensive understanding of the specific details within the material. When combined with computational mechanics, this approach allows us to predict the structural response through numerical simulation, thereby avoiding the high experimental costs. In this study, the tensile cracking behavior of carbon–silicon carbide (C/SiC) composites is numerically simulated using the bond-based peridynamics model (BB-PD), which is based on geometric models derived from segmented images of three-dimensional (3D) CT data. To obtain results efficiently and accurately, we adopted a deep learning-based image recognition model to identify the kinds of material and then the pixel type that corresponds to the material point, which can be modeled by BB-PD for failure simulation. The numerical simulations of the composites indicate that the proposed image-based peridynamics (IB-PD) model can accurately reconstruct the actual composite microstructure. It can effectively simulate various fracture phenomena such as interfacial debonding, crack propagation affected by defects, and damage to the matrix.

## 1. Introduction

Ceramic-based carbon fiber composites (CMCs), such as C/SiC and SiC/SiC [1,2], exhibit outstanding mechanical properties and extensive applications in aerospace and other major equipment. These superior properties stem from their multi-scale microstructure design. Nonetheless, the microstructure of CMCs can result in intricate failure modes and mechanisms [3,4]. Thus, it is crucial to establish the correlation between macroscopic mechanical response and microscopic damage failure mechanisms of CMCs. To comprehend the damage and failure mechanisms of CMCs, it is essential to elucidate their internal microstructure. The composition of these composites is intricate. Although the arrangement of woven fibers exhibits some regularity, numerous manufacturing defects arise during the manufacturing process due to various procedural factors [5,6,7,8]. The presence of defects can disrupt the ideal woven structure of ceramic-based carbon fiber materials and significantly reduce their load-bearing capacity and crack resistance. Hence, it is imperative to reconstruct the actual geometric microstructure of CMCs to precisely predict the mechanical properties and failure modes.

High-resolution digital imaging techniques for 3D space of materials, such as X-ray computed tomography (X-ray CT), nuclear magnetic resonance, and ultrasound imaging technology, offer non-destructive and non-invasive technical solutions for industrial and life sciences. In the realm of materials science, CT technology enables the accurate restoration of micro-structural information of the object under examination. By integrating digital imaging with mechanical simulation, the mechanical response of the material can be thoroughly analyzed. These methods are applied in various fields including biomedicine, materials science, and the construction industry [9,10,11].

In the field of mechanics, CT technology reconstructs the real structure of materials and has higher accuracy than traditional CAD modeling. Subsequently, it is more accurate to evaluate the mechanical properties and failure modes of composites by CT image modeling. Streck et al. [12] incorporated CT images into simulation models and conducted high-cycle fatigue tests on aluminum die-casting alloy samples containing pores, enhancing the accuracy of predicting fault locations. Zhang et al. [13] utilized CT to obtain friction performance parameters between 3D contact surfaces within a gap, subsequently studying the sealing performance of the rough interface by establishing a contact model. Han et al. [14] used CT digital imaging to reproduce local stress to investigate the influence of internal defects on the fatigue performance of additively manufactured TI-6Al-4V materials, which confirmed that cracks were induced by inherent defects. Sherzer et al. [15] developed a lattice discrete particle model based on CT images to capture the fracture damage of concrete aggregates, resulting in crack patterns that match experimental observations. And in the same year, Sherzer et al. [16] conducted a comparative study on the structural response of concrete weight coating of offshore pipelines under different loading conditions using a lattice discrete particle model and the finite discrete element method. The approach of integrating CT digital imaging with finite element analysis is known as the image-based finite element method (IB-FEM). The challenge of IB-FEM simulation for CMCs primarily lies in the reconstruction of fiber-braided materials. Naouar et al. [17] developed a µCT finite element model, utilizing the fiber model for finite element simulation, which demonstrated that the µCT finite element model can accurately simulate the deformation of the fibers. Liu et al. [18] employed X-ray CT scanning to reconstruct the representative volume element (RVE) of 3D orthogonal braided composites. The IB-FEM model effectively replicated the fiber orientation of the 3D braided composites and then the damage mechanical model was established and implemented in ABAQUS. The study demonstrates that the specific details of fiber geometry play a pivotal role in predicting the damage and failure processes of 3D braided composites. Wintiba et al. [19] devised a voxel-based geometric reconstruction method, which transforms explicit voxel RVE geometry into implicit smooth geometry, and subsequently generates tetrahedral meshes suitable for finite element simulation. The numerical simulation results indicate that within the IB-FEM model, the voxel-based damage occurs earlier and exhibits a more random distribution. Ai et al. [20] utilized the IB-FEM model to simulate the damage of C/SiC materials, and compared the results with experimental data obtained through in situ CT to validate the accuracy of the IB-FEM method.

However, when studying the fracture behavior of C/SiC materials, the finite element method faces challenges in directly simulating the fracture process because of the discontinuity of the displacement field on the crack, which causes the breakdown of the continuity assumption, and the stress-strain field at the crack tip becomes singular. Consequently, it is difficult to capture the initiation and propagation of cracks using classical continuum mechanics (CCM). In addressing this challenge, researchers have put forward various fracture criteria to predict crack propagation and demonstrate the failure process of materials. For instance, Dugdale [21] and Barenblatt [22] introduced the concept of the cohesive zone. Tvergaard [23] applied cohesive theory to the finite element method, enabling cracks to propagate solely along the interfaces of adjacent elements. Belytschko [24] and Moës [25] proposed the extended finite element method (XFEM), which permitted the crack to propagate along any path within the element rather than solely along the element boundary. However, additional criteria are still necessary to determine the propagation of the crack. To simulate the process of materials transitioning from continuous deformation to fracture, Silling [26] proposed the theory of peridynamics (PD). Silling et al. [27] employed peridynamics to the composite material modeling by constructing fiber bonds and matrix bonds. Hu et al. [28] proposed a 3D peridynamics model to characterize the structural features of microscopic fibers and utilized periodic boundary conditions to capture the actual failure mechanism in progressive damage analysis. Additionally, Mohajerani et al. [29] employed X-ray CT scanning to model irregular rock particles and applied the peridynamics theory to reproduce mechanical phenomena such as friction and cement blockage between particles.

Therefore, in order to predict the fracture problem of CMCs, with the help of high-resolution digital image modeling, that is, IB-PD is a feasible scheme. In Zhang’s research, he used CT image modeling to simulate the fracture of woven composites based on peridynamics [30]. Moreover, the utilization of CT images for peridynamic modeling necessitates accurate segmentation of the images to precisely reconstruct the geometric structure, which corresponds to the material properties. In recent years, the advancement of deep learning has significantly enhanced the field of computer vision, particularly in areas such as image classification, target detection, and image segmentation. Utilizing the robust feature extraction capability of the deep learning method, Kopp et al. [31] employed a convolutional neural network (CNN) as the backbone network of deep learning to train synchronous irradiated CT images, thereby achieving defect classification of carbon fiber reinforced polymer (CFRP) laminates. Li et al. [32] combined deep learning methods with micro-CT technology to obtain three-dimensional parameter fields of rocks for drilling, which provides advantages in more accurately simulating the rock failure process. Badran et al. [33] performed segmentation of the fibers, matrix, and defects in fiber-reinforced silicon carbide ceramic matrix composites using CNN and in situ CT images. Additionally, Gao et al. [34] utilized the deep learning module of the commercial software Dragonfly to train the CT images of silicon carbide ceramic matrix composite specimens, successfully achieving the segmentation of matrix cracks, holes, and fibers. Song et al. [35] proposed a new ResL-U-Net convolutional neural network, which reconstructed a 3D braided structure using CT images of fiberglass-reinforced plastic (FRP) materials. He et al. [36] combined a deep learning-based image recognition model with the peridynamic method to analyze the dynamic process of crack propagation in transition-layer and dual-layer coatings in two dimensions, and evaluated their cracking resistance.

In conclusion, integrating peridynamics, capable of simulating fracture and damage problems, with CT digital images segmented by deep-learning method could potentially offer a convenient approach for exploring the mechanical response of CMCs. In this study, we initially utilized X-ray computed tomography to capture the 3D microstructure image of C/SiC composite materials. Subsequently, we employed an intelligent segmentation method based on deep learning to differentiate the fiber, hole, and matrix in the slice image, while correlating the pixel information with the specific material components. Compared to previous research, there are two areas of innovation: Firstly, in contrast to modeling from 2D images, such as those obtained through scanning electron microscopy or surface photography, which are limited to planar situations, this research incorporates 3D CT image data into the strategy. We propose an IB-PD model, effectively addressing the damage simulation problem in complex spatial structures and composites. Secondly, compared to IB-FEM, which utilizes CT images, the IB-PD model presented in this study has advantages when researching damage and fracture issues, making it more suitable for the study of composite failure behavior.

The rest of this paper is organized as follows. Section 2 provides a review of the application of deep learning methods in the field of image segmentation, including the use of the U-Net network for identifying components of C/SiC materials. Section 3 introduces the theory of peridynamics and the composite material model based on the peridynamics, driven by CT image data. Section 4 presents numerical examples in two and three dimensions to validate the proposed IB-PD model. Finally, Section 5 presents the conclusions of this study.

## 2. A Briefly Overview of the Deep Learning Method

**Figure 1:**
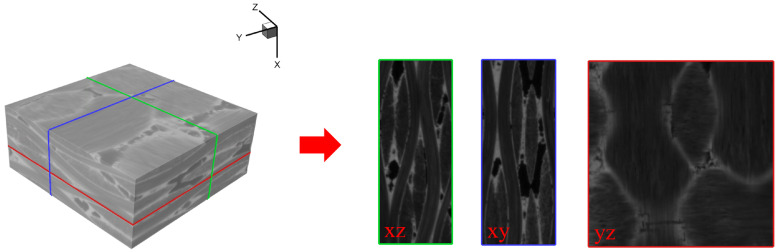
Data obtained by CT: the origin 3D CT image (**left**); slice in different directions (**right**).

In this study, image segmentation plays a crucial role in the creation of the peridynamic model utilizing CT images. Figure 1 illustrates a localized 3D CT image of C/SiC material, where various material components are distinguished by different grayscale values. Specifically, the component with the lowest grayscale value corresponds to the presence of holes, while the matrix is indicated by the highest grayscale value, and the remaining grayscale values represent the fibers. The disparity in grayscale values is attributed to the differential absorption rates of each component to X-rays. Generally, materials with higher density exhibit higher absorption rates, resulting in brighter imaging within the CT image.

**Figure 2:**
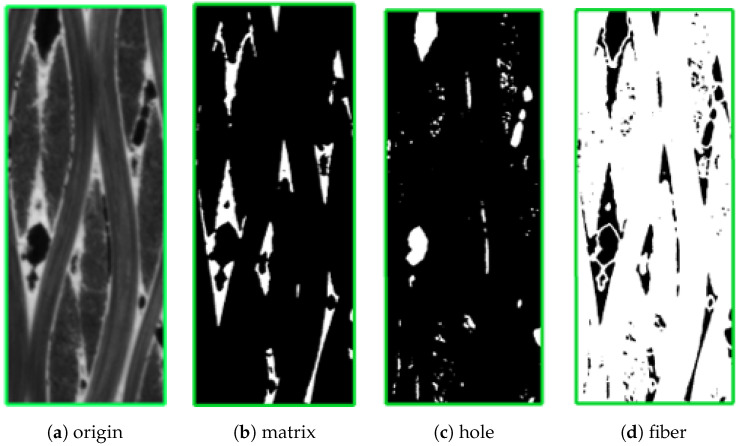
Component segmentation based on gray threshold.

The slices in three directions correspond to red, green, and blue borders as shown in Figure 1, the 3D image data can be considered as stacked with several slices in the same direction. In the segmentation of material components in CT data, the use of threshold-based segmentation methods may encounter challenges due to the small difference in gray values between the two components. It can lead to problems such as mutual inclusion and noise misjudgment. The CT slice image is threshold-segmented based on the grayscale values, and the regions corresponding to each component are displayed separately as shown in Figure 2, where the hole components are extracted separately. We can find that many areas in the fiber are incorrectly classified as holes. These small areas are referred to as *small isolated islands*. This phenomenon is caused by factors such as shooting noise and uneven material composition.

The presence of *small isolated islands* in the CT images, as demonstrated in Figure 2c,d, can introduce errors in the reconstruction of the 3D geometric model for C/SiC composites. These small isolated islands, which erroneously classify certain areas within the fiber as holes, can pose challenges in the modeling process and impact both computational efficiency and result accuracy. To address this issue, deep learning techniques have exhibited remarkable accuracy in image segmentation tasks by effectively capturing complex image features. In light of this, we employ a deep learning-based semantic segmentation method to overcome these challenges. By training a neural network model with significant depth, we can accurately segment the fibers, matrix, and holes based on their distinctive image features. This approach helps mitigate the influence of small isolated islands and noise, thus improving the overall accuracy and reliability of the segmentation process.

### 2.1. The Architecture of U-Net

**Figure 3:**
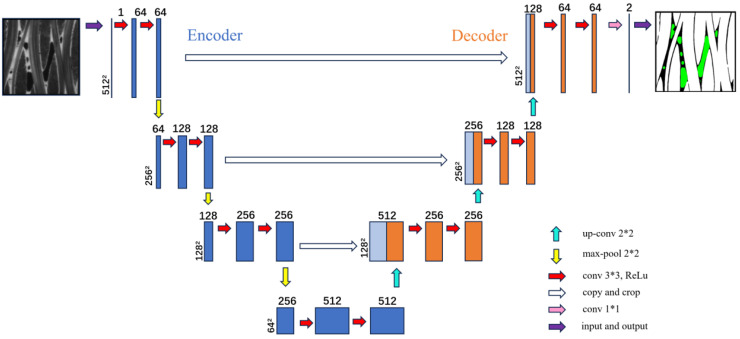
The structure of U-Net.

The deep learning model utilized in this study is the U-Net model [37], an extension of CNNs. The U-Net architecture follows a similar structure to traditional CNNs, comprising convolutional layers, pooling (downsampling) layers, and fully connected layers. These layers are recurrently employed during the construction of the U-Net model. The U-Net model adopts an encoder–decoder structure, where the encoder captures the contextual information of the image, and the decoder accurately reconstructs the segmentation results. By leveraging multiple convolution and pooling operations, the U-Net model effectively extracts pertinent features from the image and employs them for precise semantic segmentation tasks. This framework has achieved remarkable success in various domains, including medical image analysis and natural image segmentation. The unique aspect of the U-Net architecture lies in its ability to fuse low-level feature maps with rich boundary information and high-level feature maps. In this study, the U-Net network model was deployed for the segmentation of micro-CT images of C/SiC composites. The model consists of an encoder section and a decoder section. The encoder progressively reduces the feature maps, following a typical CNN architecture. Conversely, the decoder section gradually expands the feature maps. You can refer to Figure 3 for the structure of the network we employed.

The goal of training the network model is to construct a mapping function, $f_{\mathrm{pseudo},}$ such that Equation (1) holds:

(1) $$x_{CT}\left[i\right]\approx f_{\mathrm{pseudo}\;}\left(x_{CT}\left[i\right]\right)=f_{\text{U-Net}\;}\left(x_{CT}\left[i\right]\right),$$

where *i* is the corresponding position of each pixel point, $x_{m}\left[i\right]$ represents the real material composition corresponding to the location of the *i* the point, $x_{CT}\left[i\right]$ represents the original pixel gray information in the CT slice image, and $f_{\text{U-Net}}$ is the operation process of the slice graph in the whole U-Net network structure.

### 2.2. Material Composition Identification Model

**Figure 4:**
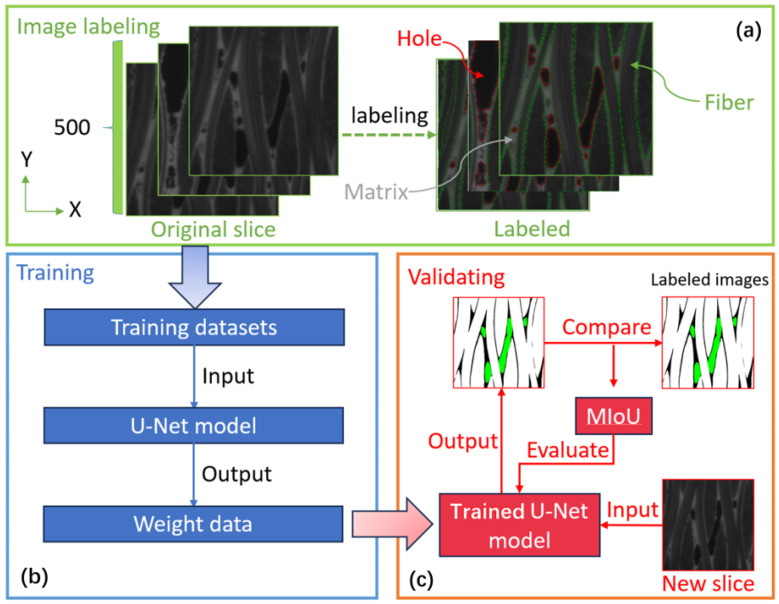
The process of training the network to identify C/SiC components. (**a**) Image labeling, (**b**) Training, (**c**) Validating.

The network was trained using a dataset comprising 500 pairs of C/SiC composite materials. Each pair consisted of an original CT slice image and a corresponding manually annotated image. The original image was acquired from the 3D CT data of the C/SiC composite material, as depicted in Figure 1 (left). To extract the original CT slice image, five sub-regions were chosen from the original data along the z-axis. The U-Net network was trained by annotating the fiber and hole components in the 500 pairs of images. The training process is illustrated in Figure 4. Specifically, the annotation map involved delineating the fiber and hole components in the original image and assigning them distinctive colors, as showcased in Figure 4a. Figure 4b shows the main inputs and outputs of the training U-Net network, which are the prepared data set and the trained network weight data, respectively. Figure 4c shows the schematic diagram of verifying the U-Net network. The accuracy of the network prediction is verified by the set of verification, and the network weight parameters are reversely optimized by the accuracy.

During the training of the deep learning network model, a loss function is employed to quantify the disparity between the predicted mask and the ground truth label region:

(2) $$\;\mathrm{Loss}\;=-\frac{1}{h\times w}\sum_{i=1}^{h}\sum_{j=1}^{w}\left\{p\left(i,j\right)\times \log\left[q\left(i,j\right)\right]+\left[\left[1-p\left(i,j\right)\right]\times \log\left[1-q\left(i,j\right)\right]\right]\right\},$$

where h,w,p denote the height, width, and probability of the label, respectively, and *q* denotes the probability of predicting the mask. This enables the iterative adjustment of the network model’s trainable parameters, aiming to minimize the defined loss function progressively until it reaches a specific minimum, indicating convergence. Since our goal is to segment materials with multiple components, the specific loss function utilized in this study is a binary cross-entropy function as proposed in [38].

When verifying, we use the mean intersection over union (mIoU) to judge the quality of network training. Intersection over Union (IoU) is the ratio of the intersection and union of the predicted value and the true value of the area occupied by a certain component in the image. mIoU is the average of IoU of all components, which is used to evaluate the segmentation accuracy of the DCNN model. The higher the mIoU, the higher the segmentation accuracy. Multi-classification segmentation can be expressed as follows:

(3) $$MIoU=\frac{1}{k}\sum_{i=1}^{k}\frac{P⋂G}{P⋃G}$$

where *k* represents the number of components of the classification, *P* represents the area of the predicted area, and *G* represents the area of the ground truth.

The model was implemented by PyTorch and trained for 100 epochs on an Nvidia 3070 GPU using the Adam optimizer, the graphics card was purchased from ASUS in China. For training, a dataset of 450 C/SiC CT slice images was utilized, with an additional 50 datasets reserved for validation purposes. The best mIoU value reaches 86.97%, and the recognition effect is shown in Figure 4c.

The reconstruction of the C/SiC geometric structure relies on transforming the two-dimensional (2D) image matrix generated by the segmentation model into a discretized model that conforms to the PD simulation. By stacking the slices in the same direction, a 3D C/SiC geometric model is obtained for PD calculations. This process can be completed within seconds, demonstrating outstanding modeling efficiency and significantly reducing manual modeling costs.

## 3. Structural Reconstruction for Peridynamic Simulation

In this section, we will briefly introduce bond-based peridynamics, discretization through meshless method, and composite material model of peridynamics.

### 3.1. Bond-Based Peridynamics

**Figure 5:**
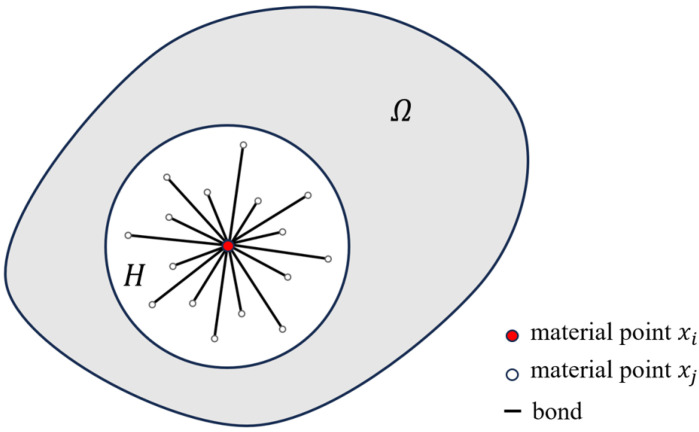
Diagram illustrating the bond-based peridynamics.

Peridynamics was proposed by Silling [26], who assumed that a point in the continuum domain Ω can interact with all points in its neighborhood through bonds as shown in Figure 5. The neighborhood of $x_{i}$ with radius δ is $H_{\delta }\left(x_{i}\right)=\left\{x_{j}\in \Omega :\left|x_{j}-x_{i}\right|\leq \delta \right\}$, where δ is referred to as the peridynamic horizon.

Based on the magnitude and direction of these forces, there are two main categories of PD formulation, namely, *bond-based* PD and *state-based* PD [39]. For the sake of simplicity, in this paper, we choose the bond-based peridynamic model, where the bonds can be seen as springs and the motion equation of bond-based PD is as follows:

(4) $$\rho \left(x_{i}\right)\ddot{u}\left(x_{i},t\right)=\int_{H_{\delta }\left(x_{i}\right)}f\left(u_{j}-u_{i},x_{j}-x_{i},t\right)dV_{x_{j}}+b\left(x_{i},t\right)$$

where ρ is the mass density, $u_{i}$ represents the displacement of point $x_{i}$ at time *t*, the dot represents the time derivative, and b represents the external body force density. The f is a pairwise force function describing the interaction between material points $x_{i}$ and $x_{j}$, and $V_{x_{j}}$ represent the volume of $x_{j}$.

For a quasi-static problem, the time-related terms are ignored, so the peridynamic equilibrium equation is obtained as follows:

(5) $$\int_{H_{\delta }\left(x_{i}\right)}f\left(\xi ,\eta \right)dV_{x_{j}}+b\left(x_{i}\right)=0$$

where $\xi =x_{j}-x_{i}$ and $\eta =u_{j}-u_{i}$. And the pairwise force function f can be expressed in terms of the deformation and the position of the bond [40], as follows:

(6) $$f\left(\xi ,\eta \right)=\mu \left(\xi ,t\right)cs\frac{\eta +\xi }{\left|\eta +\xi \right|}$$

where the stretch of bond is defined as $s=\frac{\left|\xi +\eta |-|\xi \right|}{\left|\xi \right|}$, *c* is the micro-modulus function, and μ(ξ,t) is a history-dependent scalar-valued function, which is used to describe the status of bonds, as follows:

(7) $$\mu \left(t,\xi \right)=\left\{\begin{array}{c} 1,\;\mathrm{if}\;s\left(t^{\prime },\xi \right)<s_{c},\;\mathrm{for}\;\mathrm{all}\;0\leq t^{\prime }\leq t\\ 0,\;\;\mathrm{otherwise}\; \end{array}\right.$$

where *t* and $t^{\prime }$ denote the computational time steps, and $s_{c}$ stands for a critical value script to estimate the bond state. When the bond stretch *s* is greater than $s_{c}$, which can be derived through the energy release rate $G_{0}$, the bond breaks irreversibly. For the prototype micro-elastic brittle (PMB) material used in this paper, for different cases, *c* and $s_{c}$ can be taken as follows [41]:

(8) $$c=\left\{\begin{array}{cc} \frac{12E}{\pi \delta^{4}}, & 3D\\ \frac{9E}{\pi h\delta^{3}}, & \;\mathrm{plane}\;\mathrm{stress}\;\\ \frac{48E}{5\pi h\delta^{3}}, & \;\mathrm{plane}\;\mathrm{strain}\; \end{array}\right.,\;s_{c}=\left\{\begin{array}{cc} \sqrt{\frac{5G_{0}}{6E\delta }}, & 3D\\ \sqrt{\frac{4\pi G_{0}}{9E\delta }}, & \;\mathrm{plane}\;\mathrm{stress}\;\\ \sqrt{\frac{5\pi G_{0}}{12E\delta }}, & \;\mathrm{plane}\;\mathrm{strain}\; \end{array}\right.$$

where *h* is the thickness of plane in 2D case, and *E* is the elastic modulus.

The effective damage for each point x is defined as follows:

(9) $$\phi \left(t,x\right)=1-\frac{\int_{H_{\delta }\left(x_{i}\right)}\mu \left(t,\xi \right)dV}{\int_{H_{\delta }\left(x_{i}\right)}dV}$$

which can indicate damage to the structure; when ϕ(t,x) is 0, it means that the point x is intact at time *t*, and when ϕ is 1, it means that all the bonds of the point x have failed at time *t*.

For the numerical computation of the meshless method, the pairwise potential for the bond that links point $x_{i}$ and point $x_{j}$ can be written as follows:

(10) $$\omega =\frac{c{\left(u_{j}-u_{i}\right)}^{2}}{2\xi_{ij}}V_{j}V_{i}$$

where $\xi_{ij}$ represents the distance between $x_{i}$ and $x_{j}$. Suppose that there are *k* points in the neighborhood of *i*, and the number of all points in the whole domain is *n*, so the total potential energy can be written as follows:

(11) $$\Pi =\sum_{i=1}^{n}\sum_{j=1}^{k}\left[\frac{c{\left(u_{j}-u_{i}\right)}^{2}}{2\xi_{ij}}V_{j}V_{i}-\left(f_{i}u_{i}+f_{j}u_{j}\right)\right]$$

$f_{i}$ and $f_{j}$ represent the forces on point *i* and point *j* in the local coordinate system, respectively. The matrix form of the problem can be rewritten as follows:

(12) $$\Pi =\sum_{i=1}^{n}\sum_{j=1}^{k}\left[\left[\begin{array}{cc} u_{i} & u_{j} \end{array}\right]\frac{cV_{i}V_{j}}{2\xi_{ij}}\left[\begin{array}{cc} 1 & -1\\ -1 & 1 \end{array}\right]\left[\begin{array}{c} u_{i}\\ u_{j} \end{array}\right]-\left[\begin{array}{cc} f_{i} & f_{j} \end{array}\right]\left[\begin{array}{c} u_{i}\\ u_{j} \end{array}\right]\right]$$

According to the principle of minimum potential energy, i.e., $\frac{\partial \Pi }{\partial u}=0$, the following system of linear equations can be obtained as follows:

(13) [K][U]=[F]

*U* represents the vector of the set of all point degrees of freedom, *F* represents the force vector corresponding to the direction of the degree of freedom, and *K* is the global stiffness matrix established by the whole point set according to the stiffness of the bonds. The global stiffness matrix is assembled by calculating the stiffness of each bond, as follows:

(14) $${\left[K\right]}^{b}=\frac{cV_{j}V_{i}}{2\xi_{ij}}\left[\begin{array}{cc} 1 & -1\\ -1 & 1 \end{array}\right]$$

where the superscript *b* represents a pair of bonds. In the 3D case, the local coordinates of the force and the global coordinate transformation are as follows:

(15) $$\left\{\begin{array}{c} f_{i}=\bar{f_{i}^{x}}\cos\alpha +\bar{f_{i}^{y}}\cos\beta +\bar{f_{i}^{z}}\cos\gamma \\ f_{j}=\bar{f_{j}^{x}}\cos\alpha +\bar{f_{j}^{y}}\cos\beta +\bar{f_{j}^{z}}\cos\gamma \end{array}\right.$$

where $\bar{f}$ stands as a force vector in the global coordinate system, for example, $\bar{f_{i}^{x}}$ represents the force on point *i* in the *x* direction in the global coordinate system. α,β,γ are the angles between the bond and the axis of the global coordinate. Therefore, Equation (15) can be written as follows:

(16) $${\left[f\right]}^{b}=\left[Q\right]{\left[\bar{f}\right]}^{b}$$

where

(17) $${\left[f\right]}^{b}=\left[\begin{array}{c} f_{i}\\ f_{j} \end{array}\right],\left[Q\right]=\left[\begin{array}{cccccc} l & m & n & 0 & 0 & 0\\ 0 & 0 & 0 & l & m & n \end{array}\right],{\left[\bar{f}\right]}^{b}=\left[\begin{array}{c} f_{i}^{x}\\ f_{i}^{y}\\ f_{i}^{z}\\ f_{j}^{x}\\ f_{j}^{y}\\ f_{j}^{z} \end{array}\right]$$

and l=cosα,m=cosβ,n=cosγ.

Similarly, the expression of the displacement in the global coordinate system is written as follows:

(18) $${\left[u\right]}^{b}=\left[Q\right]{\left[\bar{u}\right]}^{b}$$

Then, the stiffness matrix of bonds in the global coordinate system is as follows:

(19) $${\left[\bar{K}\right]}^{b}={\left[Q\right]}^{T}{\left[K\right]}^{b}\left[Q\right]$$

where

(20) $${\left[\bar{K}\right]}^{b}=\frac{cV_{i}V_{j}}{2\xi_{ij}}\left[\begin{array}{cccccc} l^{2} & \ldots & \ldots & \ldots & \ldots & \ldots \\ lm & m^{2} & \ldots & SYM & \ldots & \ldots \\ ln & mn & n^{2} & \ldots & \ldots & \ldots \\ -l^{2} & -lm & -ln & l^{2} & \ldots & \ldots \\ -lm & -m^{2} & -mn & \mathrm{lm} & m^{2} & \ldots \\ -ln & -mn & -mn & \ln & mn & n^{2} \end{array}\right]$$

Finally, the stiffness matrix of each bond is assembled into the global stiffness matrix, and the solution of the quasi-static problem can be solved from Equation (13).

### 3.2. Composite Material Model of Peridynamics

**Figure 6:**
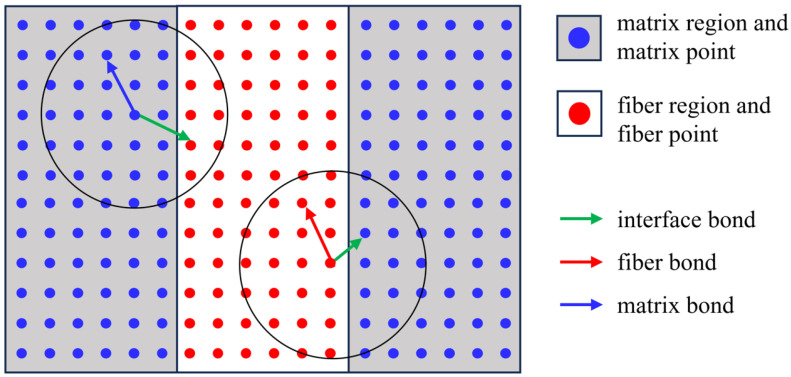
The bond relationship in fiber-reinforced composites.

Drawing upon the bond-based peridynamic theory, the analysis of composite material failure necessitates the definitions of different types of peridynamic bonds to characterize the interactions between distinct material components. Considering an idealized 2D composite plate as exemplified in Figure 6, we identify three types of bonds: fiber bonds, matrix bonds, and interface bonds. These bonds delineate the structural connections within the composite material, encompassing the interactions between fibers, the matrix material, and the fiber–matrix interface.

According to Equation (8), the micro-modulus function $c^{\alpha }$ and critical elongation $s_{0}^{\alpha }$ for each material can be determined. The properties of the interface bond are chosen as the arithmetic average between the two materials. The subscript α can be denoted as *F*, *M*, and *I*, representing the fiber, matrix, and interface, respectively.

**Figure 7:**
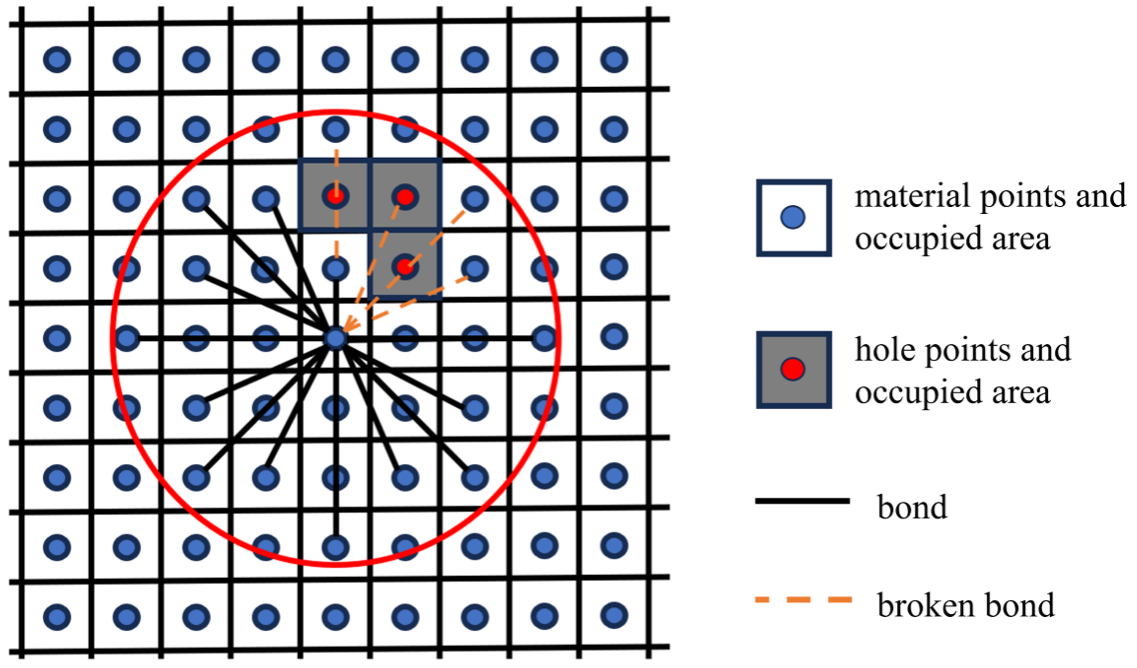
The relationship between holes and bonds in terms of shielding, the red circle represents the peridynamic horizon.

In addition to the matrix and fibers, defects are also present in C/SiC materials. These defects, manifested as holes and cracks, introduce discontinuities in the material structure. Such discontinuities diminish the impact of peridynamic bond forces. Consequently, when establishing the peridynamic composite material model based on CT images, the defect points in the image are not connected by bonds to all other material points. Prior to entering the loading phase, if a bond formed by other material points coincides with the region occupied by the hole points in space, the bond is directly disconnected, as illustrated in Figure 7. To ensure consistency between 2D and 3D analyses, we employ the slab method to determine whether a bond passes through the region occupied by a hole point. This method allows us to effectively account for the presence of defects while maintaining the structural integrity of the composite material.

**Figure 8:**
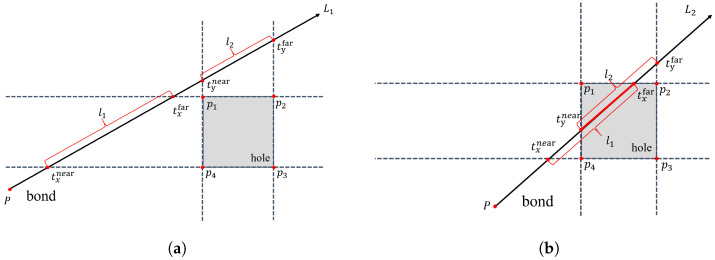
The relationship between a bond and a hole. (**a**) The bond does not pass through the hole area. (**b**) The bond passes through the hole area.

The schematic diagram for determining whether the ray passes through the region is shown in Figure 8, taking 2D as an example, consider a rectangular region on the plane that represents the area occupied by the hole point. Within this region, there are two lines, denoted as $L_{1}$ and $L_{2}$, which represent the bonds. $L_{1}$ does not pass through the rectangle, indicating the presence of a bond, while $L_{2}$ passes through the rectangle, indicating that the bond should be disconnected. It is assumed that one of the rays intersects with the extension lines of the four sides of the rectangle at points $p_{1}$, $p_{2}$, $p_{3}$, and $p_{4}$. The equation of the ray can be expressed as follows:

(21) $$L\left(t\right)=t\vec{d}+P$$

where *P* represents the starting point of the ray and *d* is the directional vector of the ray. The intersections of the ray with the horizontal and vertical lines extending from the four sides of the rectangle can be denoted as $t_{x}^{\mathrm{near}}$, $t_{x}^{\mathrm{far}}$, $t_{y}^{\mathrm{near}}$, and $t_{y}^{\mathrm{far}}$. The subscript indicates whether the intersection occurs in the *x* or *y* direction, and the superscript represents the distance from the intersection point to the starting point of the ray. Based on the intersection relationships between the ray and the four extension lines, two line segments, $l_{1}$ and $l_{2}$, are determined. These segments connect the intersections of the ray with the two extended lines in the *x*-axis and *y*-axis directions, respectively. If these two line segments overlap, it indicates that the ray passes through the region occupied by the rectangle, i.e., when we have the following:

(22) $$\max\left(t_{x}^{\mathrm{near}\;},t_{y}^{\mathrm{near}\;}\right)\leq \min\left(t_{x}^{\mathrm{far}\;},t_{y}^{\mathrm{far}\;}\right)$$

In the context of modeling, if the line segments $l_{1}$ and $l_{2}$ overlap, it signifies that the bond passes through the region occupied by the hole and becomes disconnected. Similarly, this concept can be similarly extended to the 3D case, as follows:

(23) $$\max\left(t_{x}^{\mathrm{near}\;},t_{y}^{\mathrm{near}\;},t_{z}^{\mathrm{near}\;}\right)\leq \min\left(t_{x}^{\mathrm{far}\;},t_{y}^{\mathrm{far}\;},t_{z}^{\mathrm{near}\;}\right)$$

In the 3D case, the line segment representing the bond intersects with the cube that represents the hole space. This intersection indicates that the bond in the 3D case is disconnected. By applying this method consistently, it is possible to identify and shield the bonds that pass through the hole points in a unified manner.

### 3.3. Image-Based Peridynamics (IB-PD)

In order to accurately predict the mechanical properties of C/SiC composites, it is crucial to model the entire structure. However, the C/SiC structure exhibits a high degree of complexity, with the fibers arranged in a staggered pattern in space, as well as the presence of distributed irregular holes and defects within the matrix. Constructing a full-scale specimen model involves a huge number of material points, which often results in computational cost bottlenecks. In this study, our objective is to develop a peridynamic composite material model based on CT images. To achieve this, we selectively sampled a representative region from the full-scale CT data in order to simulate the internal damage progression of the complete structure. By focusing on this smaller, yet representative area, we can effectively capture and analyze the damage process within the entire C/SiC structure.

In image analysis and computer graphics, the arrangement of pixels in a 2D image or voxel points in a 3D dataset commonly follows the Cartesian coordinate system. For a 2D image, each pixel is directly associated with a material point in the coordinate system. The pixel values correspond to different material properties represented within the image. In the case of 3D data, such as CT scans, they are typically obtained as a series of 2D slice images that have been stacked together. The voxel points in the 3D CT data are assigned to their respective material points based on the specific slice layer they pertain to. This mapping enables the reconstruction of 3D structures along with their corresponding material properties.

When working with CT data, it is important to consider the difference between pixel distances in the image and distances between real material points in 3D space. To address this, a scaling coefficient is introduced to establish a correspondence between pixels (in 2D) or voxel points (in 3D) and the actual points in the physical 3D space. By sampling the pixels or voxel points and associating them with their corresponding positions in the 3D space, the CT data can be effectively utilized for various 3D analysis and visualization purposes.

The mapping relation between the corresponding coordinates can be expressed using the following formula:

(24) $$P_{PD}\left(x,y,z\right)=f_{sample\;}\left[r_{CT}\cdot P_{CT}\left(x,y,z\right),\alpha \right]$$

**Figure 9:**
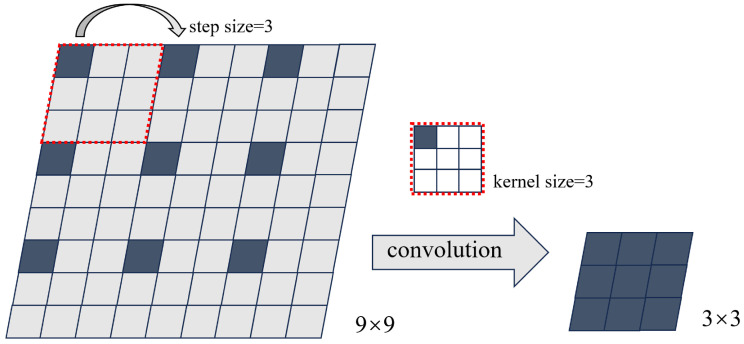
The downsampling process with a step size of 3 and kernel size of 3 × 3.

Here, $P_{\mathrm{CT}}$ (CT points) represents the coordinates of the pixels in the CT image, which are dimensionless. $P_{\mathrm{PD}}$ (PD points) represents the location of material points in the PD model, and $r_{\mathrm{CT}}$ represents the resolution of the CT image. The downsampling of the coordinates can be achieved using a convolution operation, denoted by the function $f_{\mathrm{sample}}$. This downsampling operation is illustrated in Figure 9. The downsampling coefficient α corresponds to the size of the convolution kernel and the step size used for downsampling. Note that in the context of CT imaging and the IB-PD model, the term “PD points” refers to a point in the physical space, and the “size of the resolution” refers to the voxel size or pixel size, depending on whether it is a 3D or 2D image.

**Figure 10:**
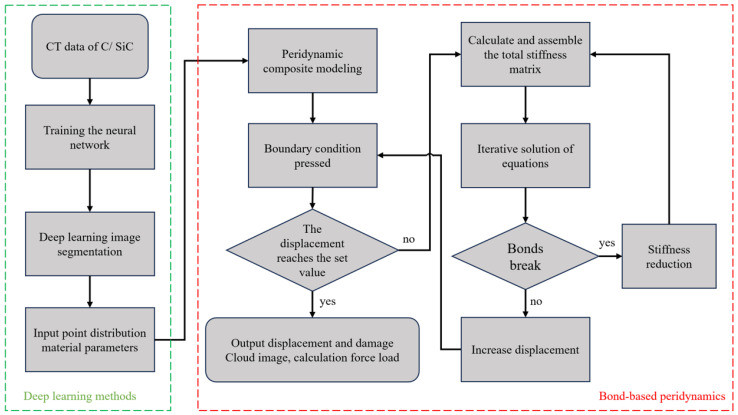
The flowchart of the numerical algorithm for C/SiC damage simulation.

The main computational process, as depicted in Figure 10, involves several steps. Firstly, the CT data slice images are manually annotated, and a U-Net network is trained to accurately distinguish the fiber, matrix, and hole components in the C/SiC material. Next, the pixel CT points are associated with the corresponding PD points. Material properties are assigned to the points, and boundary conditions are applied. The PD model is then established and solved to simulate damage and fractures, typically by assessing whether a critical failure criterion has been met.

To enhance computational efficiency, the implementation code of the IB-PD model is written in CUDA C, enabling the utilization of GPU devices. GPU acceleration is applied during the deep learning and iterative solution stages of the peridynamics equations. With the ongoing advancement of hardware capabilities and parallel computing technology, leveraging GPU or CPU/GPU hybrid computing methods holds the potential to significantly enhance the computational efficiency of the peridynamics model. This allows it to more effectively meet the requirements of practical engineering applications.

## 4. Numerical Results

In this section, we conducted simulations to analyze the tensile failure behavior of C/SiC in both 2D and 3D scenarios. The material parameters used in the simulations were obtained from the [20]. According to this reference, the elastic modulus ($E_{f}$) of the fiber is 230 GPa, and the critical fracture energy release rate ($G_{f}$) is 5 J/m^2^. The elastic modulus of the matrix ($E_{m}$) is reported as 100 GPa, with a critical fracture energy release rate ($G_{m}$) of 1 N/m^2^. The properties of the interface were calculated using the arithmetic average. Additionally, the resolution of the CT device used in the experiment was set at $1\times 10^{-6}$ m. The material property parameters used in the simulation are shown in Table 1.

### 4.1. Uniaxial Tensile Analysis in a 2D Case

**Figure 11:**
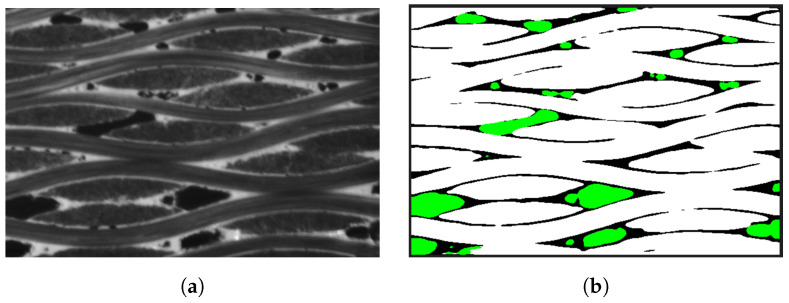
CT data and deep learning segmentation results of C/SiC. (**a**) A 2D CT image slice of a C/SiC sample (hole in black, fiber in gray, matrix in white). (**b**) The deep learning segmentation results of C/SiC samples (hole in green, fiber in white, matrix in black).

**Figure 12:**
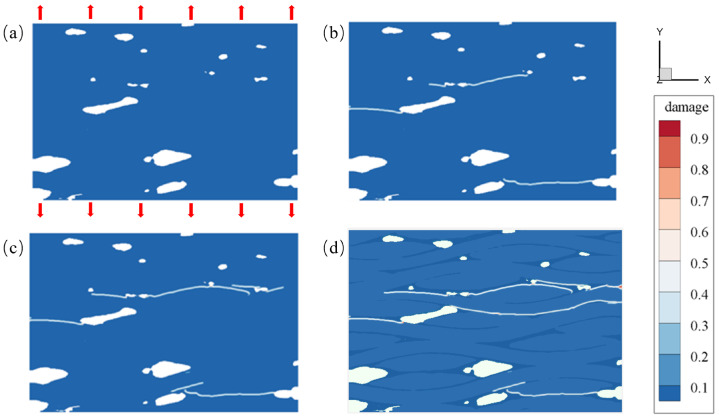
The 2D damage diagram of C/SiC composites under a tensile load. (**a**) step1, (**b**) step2, (**c**) step3, (**d**) step4.

In this section, we investigate the behavior of C/SiC materials subjected to uniaxial tensile loading. The bond-based peridynamic model satisfies plane stress conditions, with Poisson’s ratio limited to 1/3. The CT image of the C/SiC material is captured, and an original image with a size of 800 × 1200 pixels is extracted, as shown in Figure 11a. The 2D case corresponds to 0.8 × 1.2 ${\mathrm{mm}}^{2}$. Downsampling is performed with a scaling factor, α, of 2, resulting in the number of material points being 400 × 600. The trained U-Net network is utilized to classify the composition of the material, resulting in a segmentation map where different colors represent the various material components, as shown in Figure 11b. Carbon fibers are represented by white, the silicon carbide matrix is represented by black, and holes are represented by green. During computation, pixels corresponding to the holes are excluded, and the connections of bonds passing through the holes are shielded. The boundary conditions are applied along the y-axis direction, and the displacement value for each step is $2\times 10^{-6}$ mm applied on the top and bottom surfaces. The damage results at the loading step where the primary fracture occurs are depicted in Figure 12a. As the loading progresses, it can be seen from Figure 12b,c that the damage originates from the hole tip and cracks are formed to spread along the interface. To facilitate the comparison of crack initiation and propagation paths, the damage map of the final complete fracture is overlaid on the segmentation map and the original image. One of the images is made transparent and transformed, as shown in Figure 12d, allowing for clear visualization of the damage and material structure.

**Figure 13:**
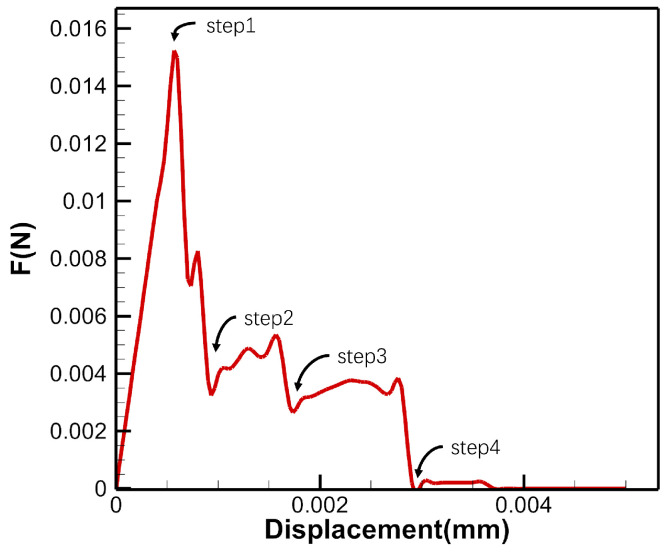
Force-displacement curve in the 2D case.

**Figure 14:**
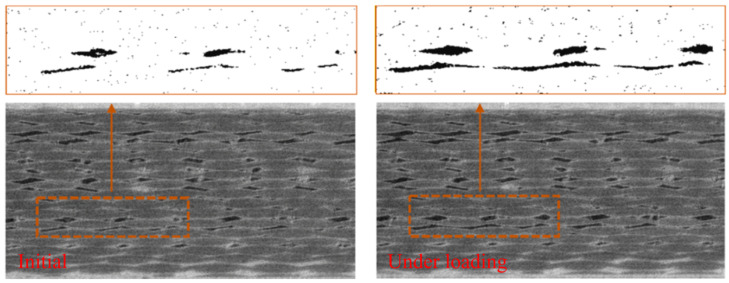
In situ tomography of C/SiC composites under a tensile load [20].

Figure 13 shows the force and displacement curves under loading in the 2D case. The thickness is calculated as 1 pixel ($1\times 10^{-6}$ mm), resulting in a relatively small load value. The stiffness reduction phenomenon due to fracture is clearly observed in the curves. The steps 1, 2, 3, and 4 correspond to stages (a), (b), (c), and (d) in Figure 12, respectively. As shown in Figure 14, the obtained results exhibit strong agreement with the in situ CT experiment of uniaxial tension conducted on a C/SiC specimen as documented in [20]. The experimental images include both the CT image and the binary image. Prior to applying the load, the composite material shows the presence of void defects. Comparing the initial structure to that under tensile load, it is evident that the specimen experiences more damage. Notably, the binary image within the yellow frame reveals the interconnection of these defects under the load, resulting in the formation of multiple intermittent cracks. Remarkably, one of these cracks nearly extends continuously throughout the entire specimen. This observation aligns precisely with the findings obtained through simulation using the 2D IB-PD model, which validates the model’s capability to simulate C/SiC composite failure.

### 4.2. Uniaxial Tensile Analysis in a 3D Case

In this section, the 3D IB-PD model is employed to investigate the damage evolution characteristics of braided C/SiC composites under uniaxial tensile load. The original CT data of the composite material is shown in Figure 1 (left). To reduce computational complexity, the CT data are downsampled. For the 3D example, downsampling is performed with a scaling factor, α, of 10, the number of pixels is 40 × 100 × 100, with the actual dimensions being 0.4 × 1 × 1 ${\mathrm{mm}}^{3}$. The U-Net network model is first utilized to intelligently segment each sliced CT image. The segmented images are then stacked together to generate the discrete peridynamic point positions and corresponding material property information in 3D.

**Figure 15:**
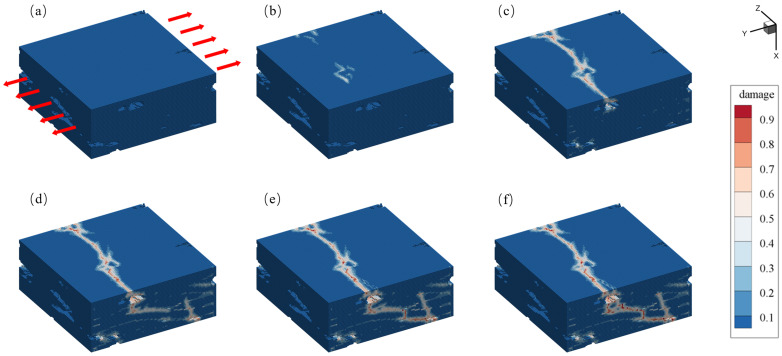
The 3D damage diagram of C/SiC composites under a tensile load. (**a**) original state, (**b**) crack initiation, (**c**–**e**) crack growth, (**f**) failure stage.

During the loading process along the Y-axis direction, the tensile displacement load is applied to the sample at the outermost three-layer material points. The displacement value for each step is $5\times 10^{-4}$ mm. The main damage diagram obtained during the loading steps is shown in Figure 15. The damage value represents the extent of damage experienced by each material point. A damage value of 0 indicates that the material point remains intact, while a value of 1 suggests that all bonds connected to the material point are broken. However, it is rare for particle damage to reach a value of 1, as this would indicate that all bonds with the relevant points have broken, meaning that the particle has exited the computational domain during loading, which does not occur in quasi-static solutions.

Figure 15 illustrates the damage process of C/SiC at different stages under loading. In Figure 15a, the initial intact geometric model is shown, with a displacement load applied in the Y direction. As loading progresses, a visible white damage region appears at the moment depicted in Figure 15b, where a significant number of peridynamic bonds between material points begin to fracture. At the moment depicted in Figure 15c, the initially appearing damaged region has developed into a crack that penetrates the surface, reflecting the process of crack propagation due to accumulated damage. In Figure 15d–f, the crack continues to extend from the surface into the interior of the material, further deepening the damage level. Ultimately, the material completely fails, as indicated by the overall structure being divided into two parts by the crack.

**Figure 16:**
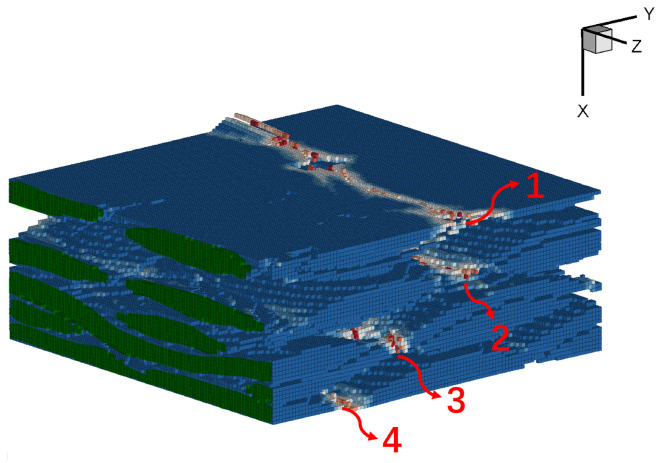
The failure result of fiber composition, 1 and 4 represent outer fiber breaks, 2 and 3 represent internal fiber breaks.

The output fiber composition is shown in Figure 16. For better visualization, the fiber section at the loading end is rendered in green, and the displacement is amplified by a factor of 100 to observe the cracking phenomenon. Due to different weaving directions, the carbon fiber cross-section mainly exhibits two shapes: spindle and ribbon, representing the interface of the warp and weft, respectively. It can be observed that fiber damage primarily occurs at the interface connected with the matrix, while damage within the fiber itself is minimal. In Figure 16, the fracture points labeled as 1–4 correspond to the weaker regions of the fiber bundle parallel to the Y-axis. These points experience gradual fracture as the loading progresses, leading to a further decrease in their bearing capacity.

**Figure 17:**
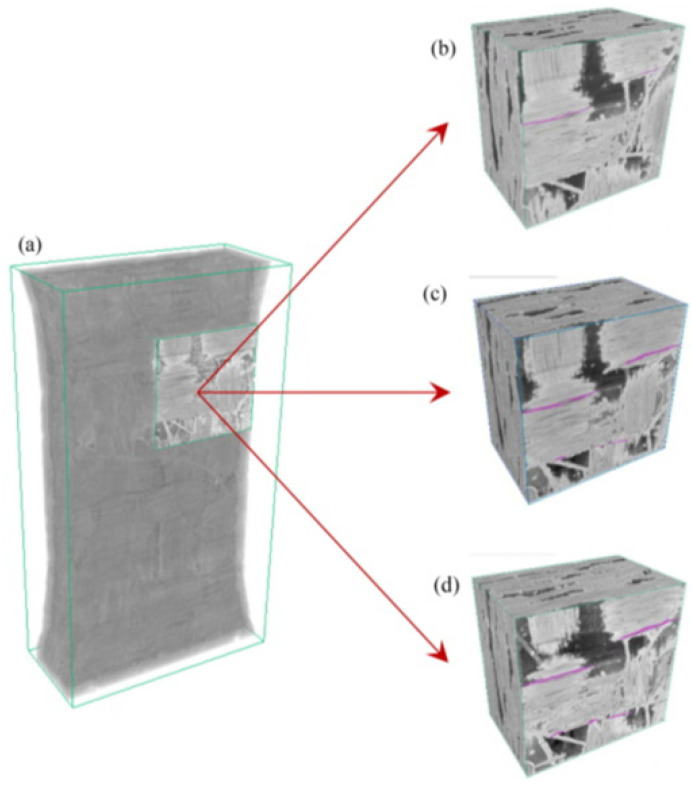
Crack evolution process of braided composite in situ CT tensile test [42]. (**a**) local specimen display, (**b**–**d**) failure process.

To better understand the damage evolution process of plain woven composites under tensile load, a reference [42] provides valuable insights through real experiments. Figure 17 in [42] illustrates the damage evolution process of woven composites, providing visual information and experimental observations of the damage progression. The visual analysis software Dragonfly is used to reconstruct and visualize the internal damage. The representation of the surface fiber bundle and matrix cracking was indicated by the color pink. By comparing the damaged region on the top surface in Figure 16 with the pink areas representing cracks in Figure 17b–d, it can be observed that the failure modes in the simulation results are consistent with the experimental findings. The stress concentration caused by the voids leads to the initiation of cracks in the surrounding material, which gradually connect with the voids as loading progresses, ultimately penetrating through the entire material region.

Furthermore, the IB-PD model, incorporating holes, is reconstructed based on the actual microstructure of the material. This model not only captures the realistic arrangement of the fiber bundles but also includes complex material defects. During mechanical loading, the evolution of material damage is a gradual process rather than an abrupt occurrence. The presence of defects leads to stress concentration, causing material damage to initiate at lower load levels. This gradual evolution of damage reflects the realistic behavior of the material under mechanical loading conditions.

### 4.3. Numerical Experiments with Different Porosity

**Figure 18:**
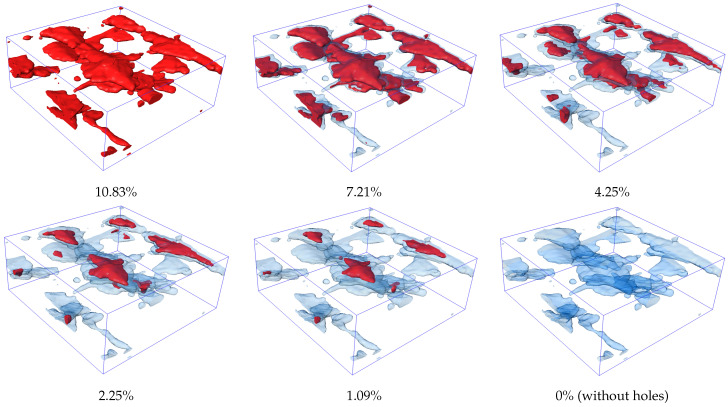
The defect distribution map under different porosities (with defects represented as red areas and the corroded regions displayed in transparent blue).

In the previous section’s simulation results, it is evident that the damage originates around the holes, with cracks initiating from the hole sites. Therefore, this subsection conducts numerical experiments with varying porosities based on the preceding numerical simulations to investigate the effect of porosity on material properties. The volume fraction of the holes was calculated using AVIZO 2022.2 software, with the porosity being approximately 10.83% in the previous simulations. Using morphological operations, the corresponding regions of the holes were eroded, and it was assumed that the eroded areas were replaced by the matrix material, resulting in a C/SiC geometric model with different porosities. The hole distributions for each group are shown in Figure 18, with porosity rates of 10.83%, 7.21%, 4.25%, 2.25%, 1.09%, and an ideal case with no internal defects. In the figure, the red areas represent the holes, while the light blue transparent areas indicate the regions where the holes have been eroded.

**Figure 19:**
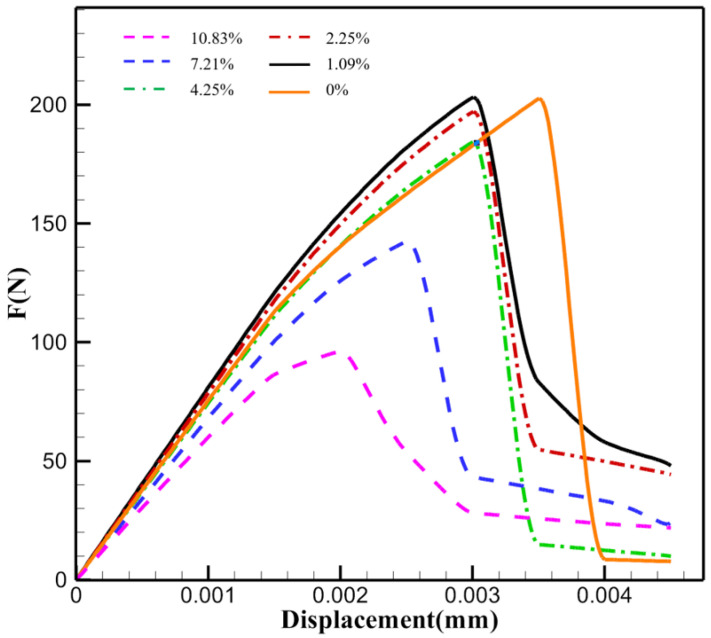
Force-displacement curves at different porosities.

For the six groups of numerical experiments, the same boundary conditions and loads as those set in Section 4.2 were applied. The load-displacement curves were generated, and the influence of porosity on the mechanical performance of C/SiC was analyzed. As shown in Figure 19, the results from six sets of numerical experiments indicate that the three groups with low porosity (porosity less than or equal to 2.25%) have a nearly identical ultimate bearing capacity, approximately 200 N. In contrast, the three groups with higher porosity exhibit a significant decrease in ultimate bearing capacity as the porosity increases; for example, the group with a porosity of 10.83% has an ultimate bearing capacity of only 96 N. It is noteworthy that there are significant differences in the critical displacement at which obvious fracture behavior occurs among the different groups. When a displacement load is applied, groups with higher porosity exhibit earlier fracture onset. Once the porosity drops below 4.25%, failure tends to concentrate within the same loading step. The analysis indicates that when porosity is 4.25% or higher, it has a significant impact on the material’s load-bearing capacity, while its influence diminishes when the porosity is below 4.25%.

Furthermore, the group with an idealized porosity of 0 exhibits a faster rate of stiffness degradation, represented by the slope, before the displacement reaches 0.003 mm, compared to groups with low porosity (2.25% and 1.09%). This phenomenon occurs because, during the initiation of cracks, if they merge with initial defects, the deformation caused by excess porosity alters the original tendency of crack propagation. This indicates that composites, compared to completely dense conditions, with a small amount of porosity can more effectively hinder the rapid growth of cracks. As a result, although the critical displacement for fracture in the group with 0 porosity is the highest, its load-bearing capacity drops to the lowest among all six groups after the fracture occurs. This suggests that an appropriate level of porosity can help resist certain brittle fracturing, preventing immediate failure when significant fracture behavior occurs. Moreover, maintaining an appropriate porosity may also delay the degradation of stiffness in composite materials before failure.

The above observations indicate that the image-based peridynamic model proposed in this study has significant advantages in analyzing the effects of various components on the material’s bearing capacity. It shows excellent potential for guiding material manufacturing processes and enhancing material performance.

## 5. Conclusions and Discussion

The IB-PD model proposed in this study has advantages in simulating materials with complex structures. The three-dimensional model improves the accuracy of the distribution of various constituents within the structure compared to the two-dimensional model. However, the computational load is a weakness of the IB-PD model. Although GPU parallel technology was employed in this study to enhance solving efficiency, the scale is still limited to local, small areas. For structural-scale simulations, we plan to couple the PD method with finite elements. In future work, we consider using the PeriFEM method to enhance the IB-PD model for more accurate failure simulations of composite materials and promote the integration of the IB-PD model with the IB-FEM model into a unified framework. In summary, the study of C/SiC composites based on the CT image data-driven peridynamics method can accurately reconstruct the geometric model of raw materials, accurately express the initial manufacturing defects of composites, and reveal the deterioration effect of manufacturing defects on the service effect of composites. The main results of this study are as follows:

(1) Using the method of deep learning to segment the components of CT images can accurately distinguish the corresponding material components of the image while providing a high-precision geometric model for simulation.
(2) The peridynamic model driven by image data can effectively establish a real geometric model and has a good effect on the internal structure characterization and failure simulation of composite materials.
(3) Manufacturing defects in C/SiC composites significantly affect their bearing capacity, with the primary failure mode being cracking along the matrix at matrix-wrapped holes.

## Figures and Tables

**Table 1:** Material parameters.

	$E_{f}$ (GPa)	$E_{m}$ (GPa)	$G_{f}$ (J/m^2^)	$G_{m}$ (J/m^2^)	Size	Fiber Fraction	Matrix Fraction
Case 2D	230	100	5	1	0.8 × 1.2 mm^2^	80.70%	13.16%
Case 3D	230	100	5	1	0.4 × 1 × 1 mm^3^	70.26%	18.90%

## Data Availability

The original contributions presented in the study are included in the article, further inquiries can be directed to the corresponding author.
